# The dose limits of teeth protection for patients with nasopharyngeal carcinoma undergoing radiotherapy based on the early oral health-related quality of life

**DOI:** 10.1515/med-2023-0673

**Published:** 2023-03-30

**Authors:** Jing Yang, Liping Yang, Qian Han, Yangyang Zhang, Zhenchao Tao, Yan Zhou, Peng Zhang, Ru Wang, Bin Sun, Jian He, Jin Gao

**Affiliations:** Department of Radiation Oncology, The First Affiliated Hospital of USTC, Division of Life Sciences and Medicine, University of Science and Technology of China, Hefei 230031, Anhui Province, China; Department of Stomatology, The First Affiliated Hospital of USTC, Division of Life Sciences and Medicine, University of Science and Technology of China, Hefei, Anhui, China; Department of Radiation Oncology, The First Affiliated Hospital of USTC, Division of Life Sciences and Medicine, University of Science and Technology of China, No. 107 Huan Hu East Road, Shushan District, Hefei 230031, Anhui Province, China

**Keywords:** nasopharyngeal carcinoma, RT, radiation-related teeth damage, caries, dose limits

## Abstract

Radiation-related teeth damage is a common complication in nasopharyngeal carcinoma (NPC) patients undergoing radiotherapy (RT) that seriously affects their oral health-related quality of life (OHRQoL). However, few studies have focused on protecting teeth function. This study aimed to calculate dental dose limits based on OHRQoL. Analysis was performed on 96 NPC patients who received RT (all received routine pre-radiotherapy dental interventions in our department). Based on the General Oral Health Assessment Index (GOHAI), OHRQoL was assigned into poor (<46) and good condition groups (≥46). The binary logistic regression analysis model was used for single-factor and multivariate analyses to identify the independent factors affecting OHRQoL. The cut-off value of dose received by teeth was obtained by drawing a receiver operating characteristic curve. NPC patients experienced a decline in OHRQoL following RT (*P* < 0.05). Univariate analysis of GOHAI revealed that the average dose of maxillary anterior teeth, the average dose received by the oral cavity, tumor volume (GTVnx), and liking of the sweet food all affected GOHAI (*P* < 0.05). Multivariate analysis indicated that the average dose of maxillary anterior teeth and liking sweet food were independent factors that influenced the OHRQoL of NPC patients with RT. When the average dose received by maxillary anterior teeth is greater than 28.78 Gy, and there is a tendency in sweet food, the OHRQoL will deteriorate. NPC patients who received RT had a better OHRQoL if the average dose to maxillary anterior teeth was limited to less than 28.78 Gy and the intake of high-sugar foods was reduced.

## 1 Introduction

As known, the 5-year survival rate of nasopharyngeal carcinoma (NPC) patients can exceed 80% [1]. Radiotherapy (RT) is an essential treatment option for NPC. While RT frequently cures tumors, it leaves the patient with long-term side effects. Radiation-related teeth damage is a common complication; it has been estimated that approximately 30% of patients undergoing RT for head and neck tumors develop radiation-related caries [2]. Additionally, periodontitis, teeth sensitivity, delamination, enamel cracks, teeth loss, and even jaw osteonecrosis may occur, affecting chewing, swallowing, pronunciation, and perception of food [3,4]. Treating dental fillings, implants, and jaw reconstruction are costly in all countries globally, imposing a heavy economic burden on the patient’s family. Currently, there are only a few preventive methods, such as cleaning teeth before RT, removing caries, and treating periodontitis. However, teeth damage caused by RT through pre-radiotherapy intervention has not yet been addressed. In contrast to other RT departments, teeth are routinely delineated as organs at risk for NPC patients in our department, and dose limits are given so that physicists can reduce the dose of radiation exposure to teeth according to the dose limits when making plans to prevent teeth damage.

Parahyba et al. [5] reported the dose distribution of maxillary and mandibular teeth in NPC and oropharyngeal cancer patients undergoing RT. They proposed that molars of the posterior group received higher radiation doses than others. Gawade et al. [6] discovered that teeth damage and its severity positively correlate with RT doses. Walker et al. [7] analyzed the correlation between exposure dose of teeth and teeth damage degree and found that the degree of radiation caries gradually increased as the radiation dose increased. When the radiation dose exceeded 60 Gy, it caused significant damage to the teeth. However, Epstein [8] and others believe that teeth damage is mainly due to changes in saliva volume and function. While the incidence of osteoradionecrosis is positively correlated with radiation dose. According to Zhang et al. [9], RT damage to teeth is primarily caused by the direct action of radiation and destruction of salivary glands, which reduces the saliva flow rate, decreases pH value, and indirectly promotes teeth damage. The development of precision RT technology has improved the protection of parotid gland and oral environment, decreased the incidence and severity of dry mouth, and improved indirect damage to a certain extent. However, these studies are limited to listing the doses received by teeth and are not based on oral health-related quality of life (OHRQoL), which reflects the subjective feelings of patients, and there is no research on NPC, which is a special head and neck tumor. Concurrently, the consensus on NPC guidelines does not mention the dose-volume limits of teeth. The innovation of this study was to prevent loss of dental function in NPC patients who received RT by reducing the dose received by each part of the teeth through pre-radiotherapy intervention.

Currently, there is no unified scale for OHRQoL assessment. GOHAI is a questionnaire for OHRQoL assessment that has recently become increasingly popular. It is a self-reporting tool originally called the Geriatric Oral Health Assessment Index that can be completed easily. Subsequently, it was referred to as the General Oral Health Assessment Index. The Health Assessment Index generally applies to all age groups [10,11]. It has been translated into multiple languages and is widely employed in many countries in North America, Europe, Asia, Hong Kong, and mainland China for epidemiological statistics and the assessment of patient’s oral health and quality of life. Translated into Chinese by Wang [12] and others, the Mandarin Chinese version of GOHAI has good reliability and validity following evaluation. Similar to GOHAI, the Oral Health Impact Profile-14 (OHIP-14) has been widely validated. However, it has many subjective items that are difficult to understand for people with lower academic qualifications. Ikebe et al. [13] believe that the GOHAI is more sensitive to objective values of oral functions. Tasaka et al. [14] evaluated oral QOL after IMRT for NPC using the GOHAI. In this study, the teeth were protected as a single organ at risk. After RT, patients were followed up, the number of decayed, missing, and filled teeth (DMFT) was recorded, the early and late GOHAI scores were analyzed, and statistical analysis was conducted. Furthermore, we analyzed the relationship between quality of life and radiation dose, as well as its relationship with tumor stage and location and patient characteristics.

## 2 Materials and methods

### 2.1 Study participants

The case data retrospectively analyzed NPC patients admitted to the Department of Radiation Oncology, the West Brach of First Affiliated Hospital of University of Science and Technology of China (USTC), from November 2018 to February 2021. The follow-up period was between 12 and 38 months, and the median follow-up period was 26 months. The eighth edition of the AJCC on Cancer staging system was used. Inclusion criteria were pathologically confirmed by nasopharyngeal biopsy, KPS score ≥70, age <65 years, radical RT in our department, and ≥2 functional teeth in any 1/6 functional segment of the oral cavity. Exclusion criteria were as follows: the head and neck had received RT for tumors other than NPC, combined with malignant tumors of jaws and teeth, Sjogren’s syndrome, or diabetes.


**Figure 1:**
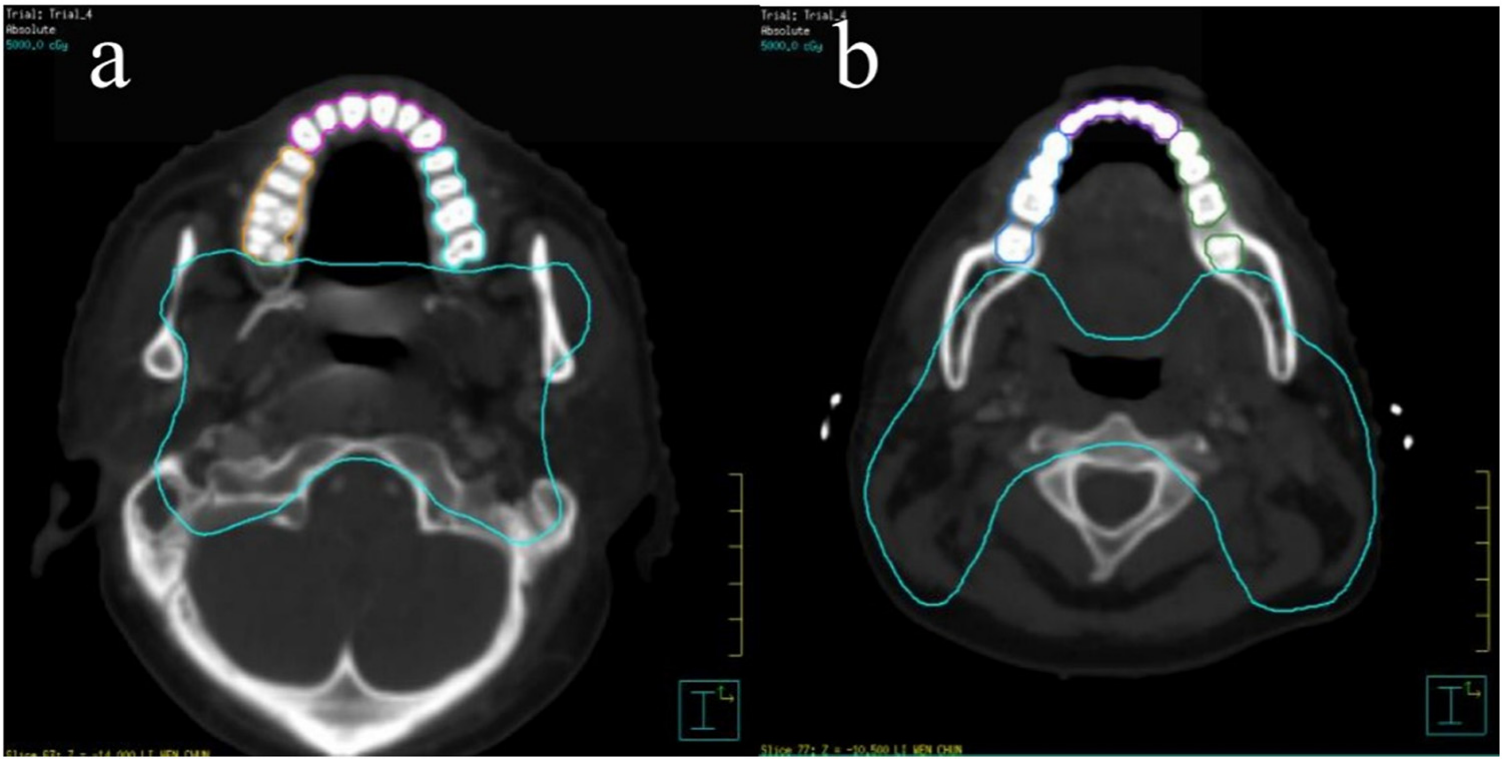
(a) Delineation of the maxillary teeth on CT images and (b) delineation of the mandibular teeth on CT images (The dose line is 50 Gy. CT: window: 1,500, level: 800.).

**Ethical approval:** This study was conducted in accordance with the Declaration of Helsinki with informed consent from the participants, and approved by the Ethics Committee of the West Brach of First Affiliated Hospital of USTC (2022-FLK-01). Informed consent was obtained from all individual participants included in this study.
**Consent to publish:** The authors affirm that research participants provided informed consent for publication of the images in Figure 1a and b.

### 2.2 Target tumor delineation and dose assessment

All patients received volumetric modulated arc therapy (VMAT), in which they were fixed in the supine position with a head-neck-shoulder thermoplastic mask and underwent RT CT simulators (GE). The scanning range was extended from the top of the skull to the carina and with 2.5 mm thick sections. Images were transmitted to a Philips Pinnacle radiotherapy planning system. Target tumor volumes were sketched on enhanced CT images according to a protocol of International Commission on Radiation Units and Measurements Reports 83. The radiation doses were 70 Gy/33 fractions to the planning target volume (PTV) of nasopharyngeal tumors and retropharyngeal lymph nodes, 66–70 Gy/33 fractions to PTV of metastatic cervical lymph nodes, 60 Gy/33 fractions to high-risk regions, and 54 Gy/33 fractions to low-risk regions, including the neck lymphatic drainage region. Tumor treatment is undoubtedly the most important, and all patients must reach the dose prescribed by the NCCN guidelines to further prevent the loss of teeth function. According to RTOG0225 and RTOG0615 reports, normal tissues were exposed to doses within the tolerable range. Using a Varian Trilogy 5339 linear accelerator, 6 MV X-ray irradiation, routinely divided, 1 time/day, 5 days/week, and kV cone-beam CT image guidance was performed weekly to detect and correct the patient’s RT position. All patients received nedaplatin (40 mg/m^2^/w) concurrent chemotherapy and completed 4–5 cycles.

### 2.3 Teeth delineation and dose assessment

According to bone window level of scan CT, the window width was 1,500, and the window level was 800. Each layer of scan CT is 2.5 mm, and teeth delineation requires approximately 7–9 layers. The maxillary and mandibular teeth were delineated separately and are divided into three groups, comprising natural teeth and dentures: right molar and premolars (maxillary or mandibular teeth-R), incisors and canines also known as anterior teeth (maxillary or mandibular teeth-A), and left molar and premolars (maxillary or mandibular teeth-L) (Figure 1). According to Liang et al. [15,16], the maximum dose of all teeth limit was less than or equal to 50 Gy; average dose limit was less than or equal to 35.8 Gy.

### 2.4 Oral health assessment

All patients were referred to a dentist at our institution before chemo radiation. Evaluation of the baseline teeth status was performed routinely. They will prophylactically remove unhealthy teeth such as caries. If the teeth are pulled out, radiation therapy cannot be initiated until 4 weeks later. No protective teeth devices were used during RT. All patients underwent follow-up dental appointments after RT. An oral health assessment was performed by the same experienced dentist using LED shadowless lights, disposable mouth mirrors, and probes based on 28 teeth. This study used World Health Organization standards to record the total number of participants’ teeth, the DMFT index, and the patient’s subjective GOHAI scale on the day of the start and end of RT. GOHAI has 12 items and three dimensions: physical function, pain and discomfort, and psychosocial function. GOHAI scores were distributed between 12 and 60. A higher score implies better OHRQoL. These indicators were evaluated before and after RT. The optimal cut-off value for the GOHAI scale remains unclear with previous studies. Many similar scales use the median or quartile as the cut-off value. If we use the quartile as the cut-off value, the number of positive patients is small, which is inconsistent with the fact that most patients in the clinical real world complain of poor oral quality of life. The median value of the GOHAI scale post-therapy was 46; Therefore, we determined the cut-off value of 46. It is defined as 1 if less than 46, indicating poor OHRQoL, and if it is greater than or equal to 46, it is defined as 0, indicating good OHRQoL.

### 2.5 Data collection and model building

All 96 NPC patients were used to record the following information: the average and maximum radiation doses received by all groups of maxillary teeth and mandibular teeth, the parotid gland, submandibular gland, oral cavity, and tumor volume (GTVnx). Various information is collected about gender, age, tumor location, number of teeth brushing frequency, sweet food liking, smoking status, TNM staging, and educational background (Table 1). The above factors were combined with GOHAI score to establish a binary logistic regression model to find out the independent influencing factors of oral health QoL. The sensitivity and 1-specificity of independent influence factor fit the receiver operating characteristic (ROC) curve to find the cut-off value.

**Table 1:** Single-factor analysis results affecting GOHAI

Characteristics	GOHAI = 0 (*n* = 60)	GOHAI = 1 (*n* = 36)	*P*
Gender			0.451
Male	38	20	
Female	22	16	
T stage			0.618
T1	2	1	
T2	28	12	
T3	23	18	
T4	7	5	
N stage			0.732
N1	21	12	
N2	28	15	
N3	11	9	
Tumor location			0.68
Left	12	10	
Right	24	13	
Non-left and right	24	13	
Teeth brushing frequency (/day)			0.784
1	20	10	
2	34	23	
≥3	6	3	
Smoking			0.564
Yes	24	12	
No	34	22	
Liking of the sweet food			0.005
Yes	16	20	
No	44	16	
Educational background			0.392
Elementary school and below	22	17	
Junior high school	25	12	
Senior middle school	5	5	
University and above	8	2	
Age (years)	47.78 ± 10.31	48.28 ± 9.92	0.686
Tumor volume (GTVnx) (cm^3^)	41.99 ± 23.04	53.39 ± 29.56	0.037
Maximum doses (Gy)			
Maxillary teeth-L	51.89 ± 4.47	52.77 ± 6.36	0.445
Maxillary teeth-A	36.66 ± 6.85	37.83 ± 5.10	0.373
Maxillary teeth-R	51.85 ± 5.56	52.30 ± 5.88	0.705
Mandibular teeth-L	45.56 ± 5.79	46.85 ± 8.41	0.37
Mandibular teeth-A	32.11 ± 6.52	32.61 ± 6.15	0.706
Mandibular teeth-R	45.87 ± 6.36	45.32 ± 7.67	0.702
Average dose (Gy)			
Maxillary teeth-L	36.04 ± 5.06	37.23 ± 5.64	0.281
Maxillary teeth-A	27.10 ± 5.94	29.66 ± 4.38	0.027
Maxillary teeth-R	35.46 ± 5.25	37.04 ± 5.89	0.172
Mandibular teeth-L	31.68±4.97	31.32 ± 4.66	0.722
Mandibular teeth-A	22.83 ± 4.87	23.70 ± 3.79	0.357
Mandibular teeth-R	29.59 ± 5.46	31.43 ± 4.46	0.089
Maxillary teeth-L+R	35.75±4.55	36.99 ± 5.31	0.226
All maxillary teeth	32.72 ± 4.72	34.34±5.05	0.113
Mandibular teeth-L+R	30.75 ± 4.01	31.32 ± 3.86	0.491
All mandibular teeth	28.33 ± 3.91	28.70 ± 3.38	0.627
All teeth	30.65 ± 3.72	31.88 ± 3.69	0.116
Parotid gland-L	30.14 ± 6.84	29.17 ± 4.95	0.455
Parotid gland-R	29.25 ± 4.53	28.49 ± 3.04	0.373
Submandibular glands-L	59.25 ± 5.66	59.47 ± 6.28	0.857
Submandibular glands-R	60.20 ± 4.81	60.60 ± 4.53	0.681
Oral cavity	36.70 ± 5.31	39.04 ± 3.92	0.024

### 2.6 Statistical methods

SPSS 17.0 was used for statistical analysis. The difference in DMFT index (dDMFT) between patients before and after RT analysis was performed using the rank sum test as data did not follow a normal distribution. The measurement data conformed to the normal distribution are represented as ¯*x* ± *s*. The paired *t*-test was used to compare the differences in GOHAI scores between patients before and after RT. The independent sample *t*-test was used to compare poor OHRQoL and good OHRQoL groups before RT. GOHAI score after RT was analyzed using binary logistic regression analysis. The variables of single-factor analysis *P* < 0.05 were introduced into the multivariate analysis model, and the independent influencing factors of GOHAI in NPC patients were screened out with *P* < 0.05. ROC curve was plotted to obtain the cut-off value of teeth limit. Differences were considered statistically significant at a threshold of *P* < 0.05.

## 3 Results

Total 96 NPC patients were included in this study, including 58 males (60.4%) and 38 females (39.6%), aged 20–65 years, with a median age of 49.5 years. General characteristics of patients are shown in Table 1. The total teeth number range is 19–32, with a median of 29. The DMFT (not everyone has the third molar, so we based on 28 teeth) range was 0−10, with a median of 2.

Here, we discuss only the acute reactions. The number of patients with apparent changes in DMFT was relatively small. Only one patient experienced post-radiotherapy complications (dDMFT = 1); the anterior teeth fell out, and no statistically significant difference was observed before and after RT (*Z* = −1, *P* = 0.317).

**Figure 2:**
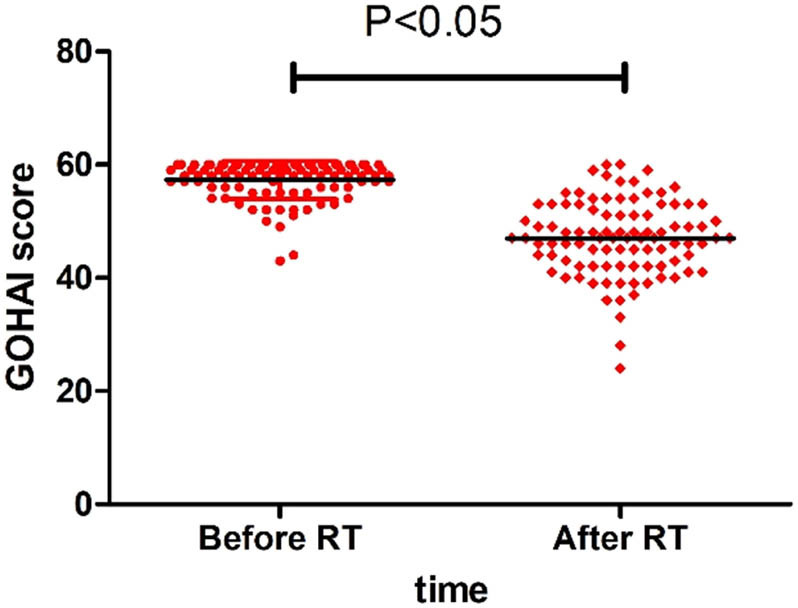
Changes of GOHAI in NPC patients before and after RT (*P* < 0.05).

The average score of GOHAI before RT was 57.29 ± 3.375 (95% CI: 56.61–57.98); whereas, after RT, it was 46.92 ± 6.669 (95% CI: 45.57–48.27). This demonstrates that the overall quality of life of teeth is acceptable. However, after RT, it is poor. As Figure 2 displays and compares the two OHRQoL scores, the comparison was statistically significant (*t* = 15.69, *P* < 0.0001), indicating that patients’ quality of life after RT was significantly reduced. Among the three sections of GOHAI, functional limitation and pain and discomfort scores were significantly reduced, while those of psychological discomfort section were not significantly reduced. About 94% of patients had varying degrees of dysphagia, and 69% had varying degrees of teeth sensitivity. Simultaneously, only 15% of patients interfered with speaking, and 22% restricted themselves from interacting with others due to dental problems.

Comparison between patients with good OHRQoL (GOHAI = 0) and poor OHRQoL (GOHAI = 1) groups demonstrated no statistically significant difference before RT (*P* > 0.05). As illustrated in Table 1, the logistic regression for single-factor analysis affecting GOHAI after RT revealed factors of *P* < 0.05, the average dose of maxillary anterior teeth group, the average oral dose, tumor volume (GTVnx), and preference for sweet food.

**Figure 3:**
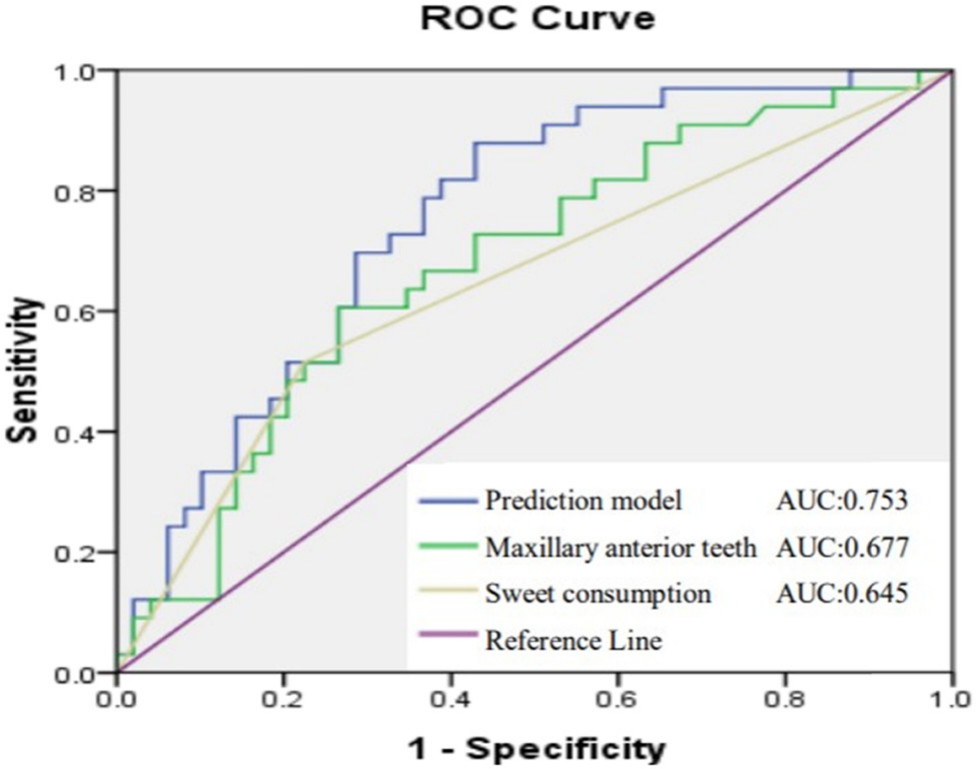
ROC curve of maxillary anterior teeth (*P* = 0.006) and liking the sweet food (*P* = 0.007).

The binary logistic regression model for multivariate analysis was fitted with a single factor analysis of meaningful factors, and the results indicated that the dose of maxillary anterior teeth and liking sweet food were independent influencing factors of GOHAI (Table 2). Area under curve (AUC) can be obtained by summing the area of each part under the ROC curve. As a numerical value, AUC can directly evaluate the quality of the classifier. The larger the value, the better the effect. As illustrated in Figure 3, the AUC value in this model is 0.753, which indicates that the logistic regression model has good prediction effect. Following RT, probability that the OHRQoL of NPC patients who enjoy sweet food will deteriorate is 4.233 times that of patients who do not enjoy sweet food. The cut-off value for the average dose of the anterior teeth of maxillary teeth affecting OHRQoL is 28.78 Gy.

**Table 2:** Multi-factor analysis results affecting GOHAI

Factors	*B*	Wald	*P*	OR (95% CI)
Mean dose of maxillary anterior teeth	1.443	7.532	0.006	4.233 (1.511–11.864)
Liking of the sweet food	0.002	7.300	0.007	1.002 (1.000–1.003)

## 4 Discussion

After RT for NPC patients in this study, most DMFT remained unchanged. According to Palmier [2] and others, radiation-related caries mainly develops 6–12 months after RT. The existence of radiation-related dental caries is associated with an increased DMFT index. In this study, only one tooth was lost after RT. It has not been observed to be statistically significant, which is believed to be due to its short duration.

This study found that GOHAI is more sensitive and that OHRQoL after RT is significantly lower than before RT. Compared to the condition of teeth before RT, the proportion of teeth sensitivity after RT increased, consistent with the findings of Kataoka et al. [17]. This reduces the cleaning time and the degree of teeth cleanliness, further deteriorating oral hygiene and increasing the chance of tooth decay. Owing to the tumor location near the patient’s mandible and posterior molars, the posterior molars are deep and difficult to clean, and dental caries are easily formed. However, the strange thing is that while the incidence of radioactive caries is highest in incisors and canines, it is lowest in posterior molars. In our study, single-factor and multivariate analyses revealed that only the average dose to the maxillary anterior teeth and liking sweet food were independent influencing factors. This can explain why radioactive caries is more likely to occur in the anterior teeth than in the molars.

Many studies [18–20] have revealed that liking sweet food demonstrated a significant inverse association with oral health and was a high-risk factor for coronavirus disease 2019 (COVID-19) and death. In clinical practice, we observed that the OHRQoL of diabetes patients was worse, so in the study, we excluded patients with diabetes. After RT, the probability of OHRQoL deterioration of patients who like sweet food is much higher, OR up to 4.233. This further explains that high-sugar diet plays a bad role in the process of RT. Sugar not only reduce the body’s immunity but also the pH value of the mouth and body. Excessive sugars consume calcium during decomposition and can easily cause teeth demineralization. We want to have a certain guiding role in the diet of NPC patients and hope to give patients professional advice to reduce sugar intake and increase protein intake during RT.

Although Liang’s [15] study revealed that the dose limits of premolar teeth should be less than 50 Gy, our study depicts that average dose of maxillary anterior teeth is closely associated with OHRQoL and is the most common site for radiation-related teeth damage. Several studies have demonstrated that a great amount of damage to the structure, hardness, and tensile strength of dentin, enamel, and the dentin enamel junction when they receive high-energy X-ray irradiation affects its mechanical properties and fracture resistance [21–23]. Consequently, a possible explanation is that the structure of the anterior teeth is thinner than that of the molars and usually responds to tensile pressure and compression. After radiation exposure, the hardness decreased rapidly, and demineralization occurred earlier. Therefore, we believe that a dose limit of 28.78 Gy for the average dose to the maxillary anterior teeth is reasonable and achievable in clinical practice.

The larger the volume of tumor (GTVnx), the greater the influence on the oral cavity and teeth that are closer to the nasopharyngeal lesion and the worse the OHRQoL. There are many small salivary glands around the oral cavity, and the radiation dose they receive is positively correlated with GOHAI. The higher the oral RT dose, the higher the reduction in the GOHAI score. However, the above two factors have not been determined independently in multivariate analysis, and the details need to be further confirmed in a larger sample study.

This study has limitations: the sample size was relatively small, and there was no micro-environmental research. However, with relatively complete baseline characteristic data and tooth dose limits, the results may provide reliable evidence for clinical application.

## 5 Conclusion

In summary, the average dose of maxillary anterior teeth and liking sweet food are two independent factors that influence the early quality of life of NPC patients undergoing RT. In the clinical delineation of the target area, attention should be paid to reducing the average dose of the maxillary anterior teeth. Limiting the average dose of maxillary anterior teeth to less than 28.78 Gy and limiting the high-sugar diet are conducive to promoting the patient’s OHRQoL. Completing RT and concurrent chemotherapy will be smoother, causing less impact on quality of life after RT. The recommended dose is realistic in times of VMAT.

## Appendix General Oral Health Assessment Index (GOHAI)

1. How often did you limit the kinds or amounts of food you eat because of problems with your teeth or denture?

□ never  □ seldom  □ sometimes  □ often  □ always

2. How often did you have trouble biting or chewing any kinds of food, such as firm meat or apple?

□ never  □ seldom  □ sometimes  □ often  □ always

3. How often were you able to swallow comfortably?

□ never  □ seldom  □ sometimes  □ often  □ always

4. How often have your teeth or dentures prevented you from speaking the way you wanted?

□ never  □ seldom  □ sometimes  □ often  □ always

5. How often were you able to eat anything without feeling discomfort?

□ never  □ seldom  □ sometimes  □ often  □ always

6. How often did you limit contacts with people because of the condition of your teeth and gums, or dentures?

□ never  □ seldom  □ sometimes  □ often  □ always

7. How often were you pleased or happy with the looks of your teeth and gums, or dentures?

□ never  □ seldom  □ sometimes  □ often  □ always

8. How often did you use medication to relieve pain or discomfort from around your mouth?

□ never  □ seldom  □ sometimes  □ often  □ always

9. How often were you worried or concerned about problems with your teeth, gums, or dentures?

□ never  □ seldom  □ sometimes  □ often  □ always

10. How often did you feel nervous or self-conscious because of problems with your teeth, gums, or dentures?

□ never  □ seldom  □ sometimes  □ often  □ always

11. How often did you feel uncomfortable eating in front of people because of problems with your teeth or dentures?

□ never  □ seldom  □ sometimes  □ often  □ always

12. How often were your teeth or gums sensitive to hot, cold, or sweets?

□ never  □ seldom  □ sometimes  □ often  □ always
